# Exploring the Role of Appraised Support in Mitigating Reverse Culture Shock Among Cross-Border Retirement Migrants

**DOI:** 10.3390/healthcare14020245

**Published:** 2026-01-19

**Authors:** Zenan Wu, Sai-fu Fung, Tianjian Pi, Zhai Wang, Yu Tian

**Affiliations:** 1 Department of Social and Behavioural Sciences, City University of Hong Kong, Hong Kong SAR, China; 2 Greater Bay Area (GBA) Research Cluster, City University of Hong Kong, Hong Kong SAR, China

**Keywords:** stress and coping theory, international retirement migration, returnee, GBA, subjective well-being

## Abstract

**Highlights:**

**What are the main findings?**

- This study innovatively investigates reverse culture shock (RCS) among Hong Kong retirees relocating to mainland China, applying stress and coping theory to explore how appraisals of social support are associated with lower loneliness.
- Focusing on 210 participants, we found that higher levels of RCS were associated with higher levels of loneliness, while support appraisal was more strongly associated with lower loneliness. Our findings further suggest an indirect association involving secondary appraisal, with variation across time since the last return.

**What are the implications of the main findings?**

- These insights inform cross-border policies to enhance social support perceptions among older adults relocating to mainland China and have global implications for improving mental health outcomes in ageing populations.

**Abstract:**

**Background/Objectives:** Cross-border retirement migration has become a global trend. However, this population from Hong Kong, with a unique status, offers valuable opportunities for multidimensional empirical research. This paper aims to apply a Stress and Coping Theory–based model to verify the presence of reverse culture shock (RCS) among them and explore how social support and its appraisal are associated with loneliness. It further examines indirect associations involving secondary appraisal within the appraisal structure. **Methods:** We recruited 210 Hong Kong seniors (aged ≥65) who had relocated to mainland China and had ever returned and surveyed them using validated scales. **Results:** Robust regression results revealed that higher levels of RCS were associated with higher levels of loneliness. Compared to social support (β = −0.04, *p* = 0.278), its appraisal had a significant negative association with loneliness (β = −0.09, *p* < 0.05). Mediation analysis demonstrated a significant indirect association involving social support appraisal, with variation across duration since the last return. **Conclusions:** With the resumption of normal cross-border travel after COVID-19, RCS is associated with subjective well-being among older returnees. Support appraisal shows a stronger association with loneliness, although this association varies by temporal context. We further propose that within the appraisal structure, secondary appraisal may be implicated in indirect associations linking primary appraisal to emotional outcomes, and that these associations vary by temporal context.

## 1. Introduction

In recent decades, international retirement has gained prominence globally, attracting attention not only from scholars but also from policymakers [1]. Similar to many developed countries and regions, the Hong Kong government encourages intranational cross-border retirement in mainland China, particularly in the Greater Bay Area (GBA), where the population of older adult migrants from Hong Kong continues to rise [2]. Due to policies, medical needs, and other individual circumstances, these cross-border retirees often find it necessary to return to Hong Kong periodically. Upon returning to their hometown after living abroad, they may experience difficulties reintegrating, a phenomenon known as reverse culture shock (RCS). However, the psychological impact of RCS on older returnees has been largely neglected. The stress and coping theory [3] explains how stress arises and how coping strategies are employed, forming the basis for this study. It posits that when older migrants return to their hometown, they evaluate their surroundings and resources, which may lead to stress. Reverse culture shock (RCS) can induce unfamiliarity, acting as stressors that contribute to depression or loneliness. This paper uses a quantitative approach to examine the impact of RCS on loneliness among older Hong Kong residents who moved to mainland China after retirement and returned to Hong Kong for events like the Chinese New Year. It also identifies effective factors for managing RCS and explores the appraisal structure within transitional stress and coping theory, a previously unexplored area.

### 1.1. Theoretical Foundation

According to stress and coping theory, individuals assess the relationship between themselves and their environment, and this appraisal significantly impacts their well-being [3]. When people perceive that their environment does not meet their expectations, they are likely to experience feelings of depression. Once they view this relationship as a stressor, they attempt to utilise their available resources to address it. Hobfoll, Freedy [4] introduced a resource-based explanation and proposed that stress arises when individuals lose resources, indicating that inadequate resources or ineffective coping strategies can also lead to stress. Similarly, Lazarus and Folkman [3] asserted that, in addition to primary appraisal, secondary appraisal pertains to the coping process and can also result in stress. Two factors contribute to stress: appraisal and coping strategy. The appraisal factor encompasses both the evaluation of the relationship between the individual and their environment (primary appraisal) and the assessment of the effectiveness of coping strategies (secondary appraisal). Lazarus and Folkman [3] further conceptualised that secondary appraisal focuses on evaluating the resources available to the individual, which can initiate the coping process. This theory has been widely applied in migration and ageing studies to investigate how social support (SS), as a coping strategy, moderates the relationship between appraisal outcomes and negative emotions [5]. A growing body of research has highlighted the dynamic interplay among cognitive appraisal, coping resources, and stress responses [6,7]. However, the influence of appraisal not only on coping strategies but also on emotional outcomes. Lazarus and Folkman [3] suggest that it is reasonable to exclude coping structures and focus on the internal mechanisms of appraisal in producing emotional outcomes. This aspect remains underexplored and will be discussed in the following sections.

### 1.2. Migrants and Returnees

The term *migrant* refers to a diverse group of individuals who relocate from one place to another [8]. The concept of a migrant is multifaceted and influenced by various factors, including intentions, origin, and socioeconomic context [9]. This study specifically examines a unique subset of older migrants known as cross-border retirement migrants [10], who choose to move across borders to enhance their later years.

As global population ageing becomes an increasingly pressing issue, coupled with the unequal distribution of resources for older adult care across different regions, international retirement migrants have drawn the attention of researchers. Miyashita, Akaleephan [11] emphasised the importance of healthcare availability and service capacity in shaping the relocation decisions of older adults. King, Cela [12] noted that European cross-border retirement migrants are keen on creating their own active ageing experiences. However, when their care needs escalated, they were compelled to return. In line with global trends, existing studies in Hong Kong have primarily concentrated on the factors influencing preferences for cross-border retirement. Chou [2] found that individuals who were white-collar workers and possessed a moderate level of education were more likely to emigrate. The study also identified that the portability of cross-border pension benefits played a significant role [13]. Nevertheless, few studies have examined their subjective well-being or feelings of loneliness. According to the Hong Kong Census and Statistics Department, the number of older residents (aged 65 and above) who typically reside in Guangdong Province rose from 89,000 (16.54%) in 2019 to 93,700 (18.89%) in 2023. According to the Chief Executive’s 2023 Policy Address, the cross-border retirement arrangement has been highlighted by the Hong Kong government as a solution to issues related to the ageing population. Hence, the study of this population group, particularly regarding their subjective well-being, deserves greater attention.

In this study, the term *returnee* refers to individuals who come back to their home society, i.e., Hong Kong, after living in the GBA. Upon reintegrating into their home society, they encounter various challenges, including economic difficulties [14], social and political issues [15], and, notably, re-acculturation [16]. These challenges affect their well-being and the process of readaptation, with some returnees even feeling compelled to migrate again [17]. When examining migrant status through the lens of stress and coping theory, researchers have found that migrants perceive unfamiliar environments as significant stressors, and negative appraisals can lead to a decline in well-being [18]. A similar pattern has been observed among returnees. Reverse culture shock (RCS) acts as a stressor that requires addressing through available resources [19]. This study focuses on older individuals from Hong Kong who relocated to Guangdong for senior care and subsequently returned to Hong Kong for various reasons, investigating the loneliness they experience upon their return. We aim to determine whether and how RCS impacts the level of loneliness among cross-border retirement migrants.

### 1.3. Reverse Culture Shock Among Older Returnees

Reverse culture shock (RCS) evolved from its parent construct, culture shock. Oberg [20] defined culture shock as comprising three key elements: anxiety, unfamiliarity, and social isolation, as the loss of familiar social interactions in a new environment can induce feelings of anxiety. Similarly, RCS is characterised by three main factors: expectation, an unchanged home, and an unchanged identity. Gullahorn and Gullahorn [16] noted that RCS arises from the expectation that returnees can return to an unchanged home while maintaining unchanged identities. However, the experience of living in a new culture reshapes their identities and worldviews [21]. Additionally, their expectations of an unchanged home often stem from communication with others or media portrayals [22]. This inevitable distortion of information leads to RCS for many individuals. RCS occurs following culture shock, meaning that cross-border migrants must first adjust to the new culture in their host country and then readjust to the emotional and psychological challenges upon returning home. According to Gullahorn and Gullahorn [16], these dual-phase adjustment processes can be illustrated as two U-curve graphs, each comprising three stages: the honeymoon phase, the initial high stage, characterised by excitement; the culture shock phase, the lowest stage, marked by confusion; and the recovery phase, the second high stage, representing adjustment. The second U-curve, representing RCS, leads to reentry stress, a phenomenon supported by existing studies. Gaw [22] found that over 50% of students returning from abroad considered loneliness and isolation to be significant issues. Previous research has provided evidence of the connection between RCS and negative emotions [23,24,25].

The impact of RCS does not necessarily depend on the duration of time spent abroad, as its effects can be both prolonged and profound [22]. In the model of the second U-curve, the initial honeymoon phase at home typically lasts only a few hours and rarely exceeds a month; however, the lowest stage can persist for several months [23]. Given the context of temporary returns home in this study, older individuals from Hong Kong are particularly susceptible to experiencing RCS upon their return. Recent findings suggest that RCS can be triggered by differences between cultures, including but not limited to lifestyle, social norms, religious beliefs, social disconnection, and values or ethical perspectives, as well as positive memories from the host country can evoke RCS [21]. Gaw [22] identified unmet expectations as a primary cause of RCS; individuals expect to find familiar elements from the host culture upon returning to their hometown, and RCS arises when these similarities are insufficient. Historically, significant cultural gaps have developed between Hong Kong and neighbouring regions in Guangdong, i.e., the GBA [26], with industrial development exacerbating the differences in certain value dimensions between Guangdong and Hong Kong [27].

Given this context, applying the lens of RCS to retired older adults from Hong Kong in the GBA may help elucidate some of the negative consequences they have experienced. In fact, RCS is often more challenging to overcome than culture shock, making it one of the most difficult aspects of the migrant experience [16,21,22]. RCS adversely affects well-being in the home country and diminishes the perceived value of the overseas experience [28]. Furthermore, a high level of RCS can lead to a decrease in overall life satisfaction [24]. Due to these negative impacts, Sussman [25] reported that nearly one-third of returnees regretted their decision to return, while approximately one-tenth expressed regret over their previous migration choices.

No one is exempt from RCS [16,22]. The concept of RCS was initially applied to returning armed forces veterans [29] and has recently gained prominence in the analysis of international students [30]. It is also important to note that RCS has been observed among scholars [16], missionaries [31], diaspora tourists [21], tourists visiting friends and relatives [32], and expatriate staff [33]. However, RCS has rarely been examined in the context of older returnees. Nonetheless, significant differences in values exist between generations [34], which shape behavioural and cultural norms. These differing norms among returnees lead to varying levels of adaptability and re-adaptability to their environments [28]. Therefore, RCS merits investigation beyond a generalised focus on older individuals.

**Figure 1:**
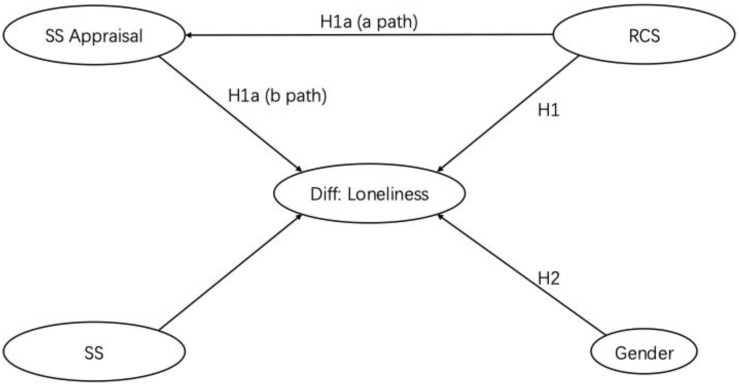
Statistical analysis model.

Numerous psychological studies have demonstrated that stressors, particularly stressful events, can manifest as loneliness in older adults [35]. Existing research indicates that older people display high levels of resilience when confronted with stressors; however, social support (SS) can influence the degree of resilience [36]. Furthermore, SS has been shown to be an effective coping mechanism for returnees facing RCS [22,37]. According to stress and coping theory, the perception of SS effectiveness significantly impacts depressive emotions. Thus, it is worthwhile to investigate the impact of RCS while controlling for SS and its appraisal results (SSA) (H1). While there is no temporal sequence between primary and secondary appraisal, both contribute independently to emotional outcomes [3]. There is a strong likelihood that appraising SS will act as a mediator between RCS and emotional responses, suggesting that secondary appraisal will be responsible for the dynamics within the appraisal structure (H1a). Both studies on RCS and stress and coping theory have reported that females tend to perform better in managing negative emotions [38] (H2). Based on the above analysis, we propose the following hypotheses concerning the cross-border retirement migrant (Figure 1):

**H1:**  *Reverse culture shock (RCS) holds a significant positive relationship with loneliness among returnees.*

**H1a:**  *Appraisals of social support (SSA) act as a mediator between reverse culture shock (RCS) and feelings of loneliness*.

**H2:**  *There are gender differences in the effects of reverse culture shock (RCS) on loneliness.*

## 2. Materials and Methods

### 2.1. Participants

Data for this study were collected using convenience and snowball sampling methods between May and June 2025, employing paper-based questionnaires. Participants were recruited from four cities in Guangdong Province of the Greater Bay Area: Guangzhou, Shenzhen, Zhuhai, and Foshan. Initial participants were sourced from retirement homes, specifically Hong Kong permanent residents who were born in Hong Kong or migrated to Hong Kong during adolescence. Based on reports from care home administrators, all participants were identified as mildly dependent, with no cognitive impairment and sufficient functional capacity to travel between mainland China and Hong Kong independently or with minimal assistance. These participants were then asked to refer others within their social networks who met the same criteria. The study aimed for a sample size of 210 Hong Kong participants, who needed to spend most of their time in China while occasionally returning to Hong Kong. Ethical approval was granted by the university’s ethics review committee, with informed consent obtained from all participants before data collection.

### 2.2. Measurement

The questionnaire was translated into Chinese using the standard procedure of back translation, with both simplified and traditional Chinese versions used to enhance understanding. Most questions and measurements were adapted from existing validated scales from published articles. The research team adapted some items from the Reentry Shock Scale to measure RCS in order to suit the context of the study. This approach ensured that the measurement tools were appropriate and reliable for the study’s objectives.

Reverse Culture Shock: The Reentry Shock Scale was developed by Seiter and Waddell [39], and is widely used among young students [22,30]. This scale contains 16 items, and each item consists of a Likert-type seven-point scale, where 1 = Strongly Disagree to 7 = Strongly Agree. A higher total score means a higher level of RCS. We translated all items into Chinese (both sample and traditional Chinese); the translated questionnaire was pilot tested on a small sample to assess clarity and appropriateness for a higher, older-friendly level and reality. We adapted some questions (changed “foreign” into “China”) and added some additional explanations for better understanding (“in China” for “host culture” and “abroad”; “in Hong Kong” for “home culture”). For the psychometric properties of the Chinese version of the Reentry Shock Scale, we evaluated the construct validity of the scale and obtained acceptable results for the CFA model fit, with CFI = 0.97, TLI = 0.96, RMSEA = 0.06 [90% CI: 0.043–0.074], and SRMR = 0.08, using the DWLS estimation method [40].

Social support appraisal: The Multidimensional Scale of Perceived Social Support (MSPSS) [41] is a self-report questionnaire that measures perceptions of SSA from different sources. There are 12 items for three dimensions (family, friends, and a significant other). Every statement is scored on a seven-point scale (1 = very strongly disagree to 7 = very strongly agree). The total score varies from 12 to 84. This scale was widely used in immigrant, older, and Chinese studies [42] and showed sufficient reliability and validity. We employed this scale to measure participants’ perception of social support in Hong Kong during their return.

Social Support: Social support was measured by the Duke Social Support Index (DSSI). The DSSI scale, developed by Duke University, is widely used for assessing older adults’ SS level. The original version consists of 35 items. Koenig and Westlund [43] abbreviate a shortened version with 23 items in three subscales. There are four items in the Social Interaction Subscale (SIS), seven items in the Subjective Social Subscale (SSS), and 12 items in the Instrumental Social Subscale (ISS). SIS and SSS were scored on a three-point Likert-type format, whereas ISS was dichotomous. The total score varies from 11 to 45, and higher scores mean more SS. This version of the scale was used to measure social support among Chinese older adults [44,45] and was measured for SS when participants returned to Hong Kong in this study.

Loneliness: Loneliness was evaluated by a three-item version of the University of California, Los Angeles Loneliness Scale (ULS-3) designed by Fritz and MacKinnon [46]. The Chinese version was verified for reliability and validity among older people. Each item was scored on a three-point scale ranging from 1 (‘Hardly ever’) to 3 (‘Often’). The three-item scale ranges from 3 to 9, with higher scores indicating a higher level of loneliness. To identify differences in loneliness resulting from the inputs, participants are asked to respond twice: once about their experience upon returning to Hong Kong and once about their experience now in mainland China.

Demographic information: This study collected some demographic information via a questionnaire. Existing studies reported that some of them were correlated with loneliness in older adults, including age, education, marital status, religious belief, presence of health problems, and length of residence. And all the above have been verified to be correlated with Chinese migrant loneliness level in older people [47]. Considering the specific background, we additionally collected information on participants’ latest return to Hong Kong (last week, last month, or last Chinese New Year), whether they had children, and the current residence location(s) of their children. This supplementary information provided comprehensive cross-border moving models and social support patterns.

### 2.3. Procedure

To examine the relationship between each variable and loneliness, we employed multiple linear regression models with heteroskedasticity-consistent standard errors (HC3 and HC1 by White [48]) to test Hypothesis 1. Given potential heteroskedasticity, this model was more reliable at obtaining trustworthy estimates [49]. HC3 estimators were applied to parsimonious models including predictors mentioned in hypotheses, whereas HC1 estimators were used for fully adjusted models incorporating a full set of demographic covariates. To examine the moderating effect of gender (H2), we created an interaction term and used the same estimation model. To examine the mediating effect of SSA between RCS and loneliness (H1a), we employed bootstrapped mediation analysis, with indirect effects estimated from 5000 bootstrap samples. For further analysis, we employed Model 14 of the PROCESS macro in SPSS 30.0 to examine whether the time since last return to Hong Kong moderated the mediating effect of SSA in the relationship between RCS and loneliness. This model specifically tested the moderation effect on the second half of the mediation path (SSA to Loneliness). A bootstrap method with 5000 resamples was used to construct bias-corrected confidence intervals for estimating indirect effects. All of the above analyses were computed in IBM SPSS 30.0 and R version 4.5.1.

## 3. Results

### 3.1. Descriptive Findings

We collected 210 valid questionnaires. All responses were from older adults over 65 years old; the sample mean was 82.20 (SD: 6.64), with a range of 65–95. The average length of residence was 3.44 years (SD: 3.28), ranging from 0.08 to 18.83 years. Other demographic factors are shown in Table 1. For further regression analysis, we handled missing data using the Multiple Imputation by Chained Equations (MICE) method for these variables [50]. The median was used for age, and the mode for education. For other categorical variables, such as health status, date of last return to Hong Kong, immigration type, and child-related items, missing values were imputed using the MICE method. RCS, SSA, SS, and loneliness were measured by Reentry Shock Scale (α = 0.87), Multidimensional Scale of Perceived Social Support (α = 0.79), Duke Social Support Index (α = 0.90), three-item version of University of California Los Angeles Loneliness Scale (α = 0.75), respectively, all scales in this study performed well in Cronbach’s alpha. Respondents reported their level of RCS (M: 66.49, SD: 12.97, range 38–103), SSA (M: 31.97, SD: 5.46, range 18–43), SS (M: 59.69, SD: 10.45, range 26–84), and delta loneliness (Pre-post difference, M: 1.08, SD: 1.51, range −2–6). In this study, we employed delta loneliness (∆ loneliness) as the dependent variable in further analysis. 

### 3.2. Regression Models

Table 2 reports findings from the regression of Hypothesis 1. In Hypothesis 1, the core variables exhibited consistent directions of association across both models, aligning with our theoretical expectations (SS: β = −0.04, *p* = 0.278; SSA: β = −0.09, *p* < 0.05). And the regression results of RCS remained statistically significant in both Model 1 (0.10; *p* < 0.01) and Model 2 (ß0.10; *p* < 0.05).

### 3.3. Mediation and Moderation Effect

We tested one mediation model and one moderation model in this study. For hypothesis 2, no evidence of gender moderation in the association between RCS and ∆ loneliness was found (β = 0.02; *p* = 0.80). We tested whether SSA statistically mediated the association between RCS (H1a) and ∆ loneliness, as seen in Table 3. In Hypothesis 1a, there was a significant indirect association between RCS and loneliness via SSA (ACME = −0.05, 95% CI [−0.084, −0.014], *p* = 0.002), a significant direct effect (ADE = 0.11, *p* = 0.006) and total effect (β = 0.07, *p* = 0.048), indicating partial mediation.

### 3.4. Further Analysis

When testing Hypothesis 1, the coefficient for SS was no longer statistically significant. Thus, we added one source of SS, their child(ren)’s location, to the original H1 model. We tried to explore whether having child(ren) residing in Hong Kong was associated with lower levels of ∆ loneliness. However, the result showed that the presence of child(ren) in Hong Kong was not significant in this model (β = 0.08; *p* = 0.281), the RCS was still performed significantly (β = 0.10; *p* = 0.022).

To evaluate Hypothesis 1a, we tested whether SSA statistically mediated the association between RCS and ∆ loneliness. We also investigated whether the time elapsed since the last return to Hong Kong moderated this mediation model (Table 4a,b). Results indicated that RCS was significantly associated with SSA (β = 0.33, *p* < 0.001), and SSA was significantly associated with ∆ loneliness (β = −0.74, *p* < 0.001). Importantly, the interaction term was statistically significant (β = 0.37, *p* = 0.017), indicating that the strength of the indirect association varied by duration. Conditional indirect associations were statistically significant when duration was short or moderate, but not when it was long.

## 4. Discussion

The results provide evidence for the presence of Reverse Culture Shock (RCS) and indicate a positive association between RCS and loneliness among cross-border older returnees. Older adults, in this study, are characteristically different and exhibit identity multiplicity. First, as older returnees, they receive senior care services in mainland China while frequently travelling between Hong Kong and the mainland for medical and other practical reasons. Their identity thus reflects the multiplex nature of circular cross-border retirement migration, characterised by both settlement and mobility across regions. The continuous redefinition of identity among circular older migrants underscores that RCS will not only occur among older people but also persists regardless of the frequency of cross-border movement (We found most seniors had more than one return experience). These two issues have not received adequate attention in prior research. In the analysis, a set of demographic variables previously shown to affect loneliness among older Chinese returnees was included (Model 2). Regression results showed that age and education level were not significant in any of the tested models. Notably, the coefficient for age was close to zero, supporting the idea that RCS is a universal experience that spares no one, regardless of age [16,22]. Although the length of stay since migration was also nonsignificant, its coefficient was negative. Three demographic indicators emerged as significantly associated with loneliness: religious affiliation, health status, and marital status. Older adults with religious beliefs reported lower levels of loneliness, consistent with previous findings [51]. After controlling for demographic factors, the significance of SS and SSA diminished. One of the possible explanations is that the commonly used demographic variables in existing literature may better reflect general older migrant populations rather than older returnees. Lastly, although previous studies [38] suggested that gender may moderate the association between RCS and emotional outcomes, our analysis did not find a significant result. This discrepancy also invites further investigation.

SS has long been regarded as an effective solution to RCS [21]. However, findings from our study suggest that it is not the objective support that plays the crucial role, but rather the subjective perception of being supported, namely social support appraisal (SSA). In the test of Hypothesis 1, only the coefficient for SSA remained significant. This indicates that for older returnees, the perception of support may be more strongly associated with subjective well-being than actual support networks. This conclusion is further supported by our subsequent analysis. In our extended H1 model, we added a variable indicating whether the respondent’s child(ren) resided in Hong Kong. However, this variable was not statistically significant, suggesting that physical proximity to support sources was not associated with loneliness in this model. This finding aligns well with the stress and coping theoretical framework. According to Lazarus and Folkman [3], appraisal structure is the primary determinant of emotional responses, while coping structure serves as a strategy mechanism, mainly aimed at emotion regulation.

The results further suggested that secondary appraisal may statistically mediate the association between primary appraisal and emotional outcomes. Within the stress and coping framework, previous research has highlighted that primary and secondary appraisal processes are not always sequential but can instead take place concurrently and interactively. Specifically, when an individual evaluates a circumstance as a stressor during primary appraisal, secondary appraisal may co-occur, jointly influencing the emotional outcomes [52]. Based on this viewpoint, we tested Hypothesis 1a, which posits that prior secondary appraisal experiences may be reflected in current appraisal patterns and be associated with how individuals appraise their present environment (primary appraisal) and report emotional states such as loneliness. Moreover, we hypothesised that primary appraisal may be indirectly associated with stress-related emotion through secondary appraisal. For instance, when previously received SS is perceived as insufficient, individuals may appraise new situations as more stressful (i.e., higher RCS). If, in such cases, their SSA is low, higher levels of loneliness may be reported. Our mediation analysis indicates a statistically significant indirect association between RCS and loneliness via SSA.

**Figure 2:**
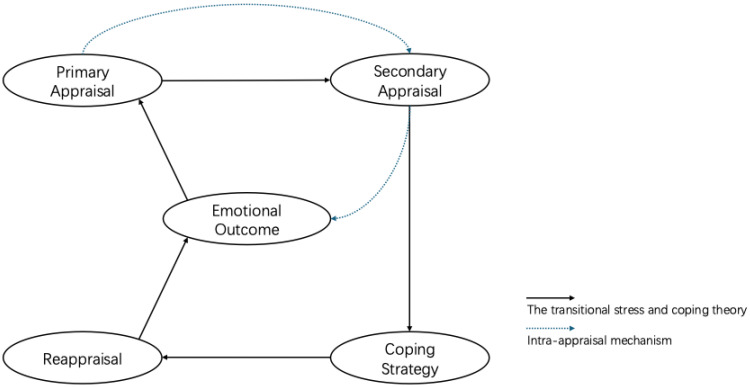
Intra-appraisal mechanism model.

In sum, our findings contribute to theoretical development by suggesting that within the stress and coping framework, primary appraisal may be associated with emotional responses both directly and indirectly through secondary appraisal (Figure 2). To the best of our knowledge, this study is among the few to examine indirect associations involving secondary appraisal within the appraisal structure, thereby adding nuance to the understanding of intra-appraisal processes in stress processing. Lazarus and Folkman [3] defined appraisal as a trigger for coping, and individuals might not apply particular strategies. Our findings suggest a multidimensional analytic perspective that may inform future empirical applications of stress and coping theory.

When the variable “duration since last return to Hong Kong” was introduced into the mediation model for Hypothesis 1a, the strength of the indirect association between SSA and loneliness varied significantly across levels of duration. While our hypothesis was originally grounded in the idea that secondary appraisal may be related to experiences surrounding the most recent return, the observed associations were weaker at longer durations since the return. This finding highlights a key nuance: although SS is widely recognised as an effective resource in mitigating RCS, associations involving SSA appear to differ by the recency of return. When perceived support is low at later stages following relocation, its association with loneliness appears weaker. Therefore, we urge policymakers to pay greater attention to returnees’ perceived social support and to maintain a high level of such perception through timely service interventions at appropriate stages.

This study is based on stress and coping theory, and our findings provide several insights that warrant further empirical examination. Firstly, due to the absence of prior research focusing on older returnees in the Chinese context, we incorporated demographic controls from studies on older migrants in Model 2. However, many of these variables produced nonsignificant results, indicating that the demographic characteristics specific to older returnees require additional investigation. Secondly, our analysis identified a previously underexplored pattern within the appraisal framework, suggesting that primary appraisal may be indirectly associated with emotional outcomes through secondary appraisal. Future research should explore this pathway using longitudinal or panel data designs. Although existing literature has addressed the interplay between appraisal and coping structures, the intra-appraisal processes by which stress appraisals influence emotional responses remain under-theorised. Lastly, we found that the time elapsed since returning to the home country was associated with variation in the indirect association involving SSA, highlighting the context of circular cross-border retirement migration. Future studies could examine whether the frequency of cross-border movements affects the severity or persistence of RCS, providing deeper insights into the relationship between migration patterns and psychological well-being in later life.

While this study offers valuable insights, several limitations must be noted. Firstly, it represents the inaugural use of the Reentry Shock Scale among older Chinese-speaking individuals. Although the confirmatory factor analysis indicated acceptable model fit indices, some items displayed borderline factor loadings, highlighting the need for better cultural adaptation and measurement refinement. Secondly, the paper-based, self-report survey format, designed for older respondents, may introduce reporting biases and result in some missing data, leading to a partial loss of analysis-ready cases; accordingly, the findings should be interpreted as associative rather than causal. Thirdly, recruiting older returnees in everyday settings is practically challenging; therefore, participants were recruited from residential care facilities. This sampling strategy may introduce selection bias related to age and health status and limit the generalizability of the findings. Fourthly, the spontaneous nature of return visits among Hong Kong cross-border retirement migrants, especially those returning regularly for chronic disease treatment, makes accurate longitudinal estimation difficult. In this study, loneliness can only be measured at some specific time points. Future research should aim to thoroughly validate the RCS scale’s psychometric properties within Chinese contexts, consider developing a version suited to the cognitive and literacy needs of older populations, and adopt it into a longitudinal study.

## 5. Conclusions

In conclusion, while the effect size of reverse culture shock (RCS) was modest, it remained statistically significant in various regression models, highlighting its influence on the loneliness of Hong Kong retirees in mainland China. With normal travel between Hong Kong and the mainland resuming fully in 2023, these older returnees are still in the early adjustment phase. Continued research is needed to assess RCS’s long-term impact on their well-being. The study suggests that perceived social support is strongly associated with lower levels of loneliness, highlighting the potential relevance of cross-border policy coordination in supporting retirees. Additionally, the findings indicate that secondary appraisal may play a moderating role within the stress and coping framework.

## Figures and Tables

**Table 1:** Descriptive Characteristics of the Sample.

Variable		Frequency	Percentage
Gender	Female	84	40%
	Male	126	60%
Education	No formal	24	11.5%
	Primary school	68	32.7%
	High school	82	39.4%
	Bachelor	33	15.9%
	Master	1	0.5%
Marital	Unmarried	1	0.5%
	Married	66	31.4%
	Divorced/separated	17	8.1%
	Widowed	126	60%
Religion	Christian	27	13.2%
	Catholic	8	3.9%
	Protestant	3	1.5%
	Buddhist	30	14.6%
	Taoist	9	4.4%
	Atheist	19	9.3%
	No religion	103	50.2%
	Other	6	2.9%
Health	Self-rated good health	79	39.9%
	hearing impairment	19	9.6%
	any chronic diseases	70	35.4%
	outpatient service or inpatient service in the last year	30	15.2%
Date of return to Hong Kong	Last week	38	18.2%
	Last month	80	38.3%
	Last Chinese New Year	90	43.1%
Number of children	0	6	2.9%
	1	35	16.8%
	2	69	33.2%
	3	57	27.4%
	4	21	10.1%
	5	14	6.7%
	6	5	2.4%
	7	1	0.5%
Child(ren)’s location	Hong Kong	88	43.3%
	Guangdong	109	53.7%
	Other places on the mainland	6	3%
	Macao/Taiwan	3	1.5%
	Overseas	25	12.3%

**Table 2:** Standardised Regression Effects.

Variable	Model 1	Model 2
Reverse Culture Shock	0.10 ** (0.05)	0.10 * (0.04)
Social support	−0.04 (0.04)	−0.03 (0.04)
Social support appraisal	−0.09 * (0.04)	−0.06 (0.04)
Constant	0.36 *** (0.03)	0.71 *** (0.18)
Age		0.02 (0.04)
Education. L		0.03 (0.10)
. Q		0.11 (0.09)
. C		0.08 (0.07)
^4		−0.00 (0.07)
Marital status (single)		−0.35 * (0.15)
Health (negative)		−0.20 * (0.09)
Religious belief (no religion)		0.20 ** (0.75)
Length of residence		−0.00 (0.04)
R. squared	0.069	0.161

*** *p* < 0.001. ** *p* < 0.01. * *p* < 0.05. Notes: Marital status (single) = Unmarried, Divorced/separated, Widowed; Health (negative) = hearing impairment, any chronic diseases, outpatient service or inpatient service in the last year; Religious belief (no religion) = Atheist, No religion.

**Table 3:** Mediating Effect.

Hypothesis		Estimate
Hypothesis 1a	ACME	−0.04 **
	ADE	0.11 **
	Total Effect	0.07 *
	Prop. Mediated	−0.54 *

** *p* < 0.01. * *p* < 0.05.

**Table 4:** (**a**) Moderated Mediation Model: Path Coefficients and Interaction Term. (**b**) Bootstrap Estimates of Indirect Effects by Moderator Level.

(**a**)							
	**Effect**	**SE**	**t**		**LLCI**		**ULCI**
RCS → SSA	0.33 ***	0.07	5.04		0.2025		0.4630
SSA → ∆ loneliness	−0.74 ***	0.22	−3.43		−1.1665		−0.3150
Interaction term	−0.37 *	0.15	2.42		0.0677		0.6696
RCS → ∆ loneliness	0.12	0.11	1.07		−0.1006		0.3383
(**b**)							
**Duration**	**Effect**	**BootSE**		**BootLLCI**		**BootULCI**	
0	−0.25	0.11		−0.4726		−0.0186	
1	−0.12	0.05		−0.2369		−0.0184	
2	−0.00	0.05		−0.0971		0.0951	

*** *p* < 0.001. * *p* < 0.05. Notes: RCS = Reverse Culture Shock; SSA = Social Support Appraisal; ∆ loneliness = delta loneliness. Notes: 0 = Last week; 1 = Last month; 2 = Last Chinese New Year.

## Data Availability

The dataset used and analysed in this study is available from the corresponding author on reasonable request.
